# Telemedicine Across the Globe-Position Paper From the COVID-19 Pandemic Health System Resilience PROGRAM (REPROGRAM) International Consortium (Part 1)

**DOI:** 10.3389/fpubh.2020.556720

**Published:** 2020-10-16

**Authors:** Sonu Bhaskar, Sian Bradley, Vijay Kumar Chattu, Anil Adisesh, Alma Nurtazina, Saltanat Kyrykbayeva, Sateesh Sakhamuri, Sanni Yaya, Thankam Sunil, Pravin Thomas, Viviana Mucci, Sebastian Moguilner, Simon Israel-Korn, Jason Alacapa, Abha Mishra, Shawna Pandya, Starr Schroeder, Ashish Atreja, Maciej Banach, Daniel Ray

**Affiliations:** ^1^Pandemic Health System REsilience PROGRAM (REPROGRAM) Consortium, REPROGRAM Telemedicine Sub-committee, Sydney, NSW, Australia; ^2^Department of Neurology, Liverpool Hospital and South Western Sydney Local Health District, Sydney, NSW, Australia; ^3^Neurovascular Imaging Laboratory & NSW Brain Clot Bank, Ingham Institute for Applied Medical Research, The University of New South Wales, UNSW Medicine, Sydney, NSW, Australia; ^4^The University of New South Wales (UNSW) Medicine Sydney, South West Sydney Clinical School, Sydney, NSW, Australia; ^5^Department of Medicine, University of Toronto, St. Michael's Hospital, Toronto, ON, Canada; ^6^Department of Epidemiology and Biostatistics, Semey Medical University, Semey, Kazakhstan; ^7^The University of the West Indies, St. Augustine, Trinidad and Tobago; ^8^School of International Development and Global Studies, University of Ottawa, Ottawa, ON, Canada; ^9^Department of Public Health, University of Tennessee, Knoxville, Knoxville, TN, United States; ^10^Department of Neurology, University Hospitals Birmingham NHS Foundation Trust, Birmingham, United Kingdom; ^11^School of Science, Western Sydney University, Sydney, NSW, Australia; ^12^Global Brain Health Institute, Trinity College Dublin, Dublin, Ireland; ^13^Movement Disorders Institute, Department of Neurology, Sheba Medical Center, Tel Hashomer, Ramat Gan & Sackler School of Medicine, Tel Aviv University, Tel Aviv, Israel; ^14^metaHealth Insights and Innovation, Manila, Philippines; ^15^Department of Anthropology, University of Delhi, New Delhi, India; ^16^University of Alberta, Alberta Health Services and Project PoSSUM, Edmonton, AB, Canada; ^17^Penn Medicine Lancaster General Hospital and Project PoSSUM, Lancaster, PA, United States; ^18^Icahn School of Medicine, Mount Sinai Hospital, Mount Sinai, New York, NY, United States; ^19^Polish Mother's Memorial Hospital Research Institute (PMMHRI) in Lodz, Cardiovascular Research Centre, University of Zielona Gora, Zielona Gora, Poland; ^20^Department of Hypertension, Medical University of Lodz, Lodz, Poland; ^21^Farr Institute of Health Informatics, University College London (UCL) & NHS Foundation Trust, Birmingham, United Kingdom

**Keywords:** telemedicine, telehealth, digital technologies, health policy, COVID-19, framework, recommendations (guidelines), geographics

## Abstract

Coronavirus disease 2019 (COVID-19) has accelerated the adoption of telemedicine globally. The current consortium critically examines the telemedicine frameworks, identifies gaps in its implementation and investigates the changes in telemedicine framework/s during COVID-19 across the globe. Streamlining of global public health preparedness framework that is interoperable and allow for collaboration and sharing of resources, in which telemedicine is an integral part of the public health response during outbreaks such as COVID-19, should be pursued. With adequate reinforcement, telemedicine has the potential to act as the “safety-net” of our public health response to an outbreak. Our focus on telemedicine must shift to the developing and under-developing nations, which carry a disproportionate burden of vulnerable communities who are at risk due to COVID-19.

## Introduction

The novel coronavirus disease (COVID-19) emerged at the end of 2019 in Wuhan (People's Republic of China) and the viral outbreak was declared as a pandemic by World Health Organization (WHO) on March 11, 2020 (1). During the conception of this article, the pandemic is still spreading, and the fight is still ongoing. The World Health Organization (WHO) COVID-19 situation report, as of September 10, 2020, showed over 27.7 million confirmed cases and over 899,000 total deaths spread across continents (2). As we continue to grapple with the “sky-scraping” deaths around the world, the difficulties of ensuring the continuity of care and maintaining the capacity of health systems pose a continued threat (3–6). The global public health response to manage and contain the pandemic has placed a paramount emphasis on telemedicine toward remote delivery of care cutting across various medical sub-specialities (7). With the exponential growth in internet users, the availability and/or accessibility of already existing telemedicine services have been bolstered. In 2018, the global telemedicine market was valued at USD 38,046 million and is expected to rise to USD 103,897 million by 2024 (8). Variations in digital preparation/maturity of hospitals, availability, access, and implementation of telemedicine are experienced across the world (9).

Pandemics challenge the preparedness of health systems-exposing the structural barriers (10). Amidst global lockdown or quarantine measures imposed due to COVID-19, access to telehealth becomes increasingly crucial and efforts to rectify disparities are warranted (11). In particular, telemedicine has a vital role to play in low- and middle-income countries (LMICs) and remote areas by improving access to healthcare to under-resourced regions (12). This article seeks to analyse the status of implementation of telemedicine around the world, and factors that influence the uptake and implementation of telemedicine. We will also discuss progress and challenges in telemedicine faced by various geographics during the COVID-19 era.

## Methods

Existing telemedicine frameworks and policies of various countries/regions and their implementation stage are analyzed using Gladhart (13) and Pan American Health Organization (PAHO/WHO) hat (14) models. An evidence synthesis approach, to summarize pre-COVID-19 and current (during COVID-19) policy frameworks and changes due to COVID-19, was undertaken to develop expert recommendations for strengthening telemedicine across the globe. Using a case-based approach, the official ministry of health or government websites, media sources, and published literature were examined to critically analyse the telemedicine frameworks and COVID-19 specific response. For each country analyzed, following policy questions were examined: (a) Is there a national telemedicine framework before COVID-19?, (b) What stage that framework is in?, (c) What are the gaps in the telemedicine implementation?, (d) What telemedicine specific policy changes, if any, have been adopted in response to COVID-19? and (e) What could be done to potentially improve the telemedicine implementation in the future in the respective country/region? Based on specific challenges identified using the analyses across geographics, specific policy-based targeted recommendations poignant to telemedicine use, during the current COVID-19 pandemic and beyond, are made.

## Results

The existing telemedicine framework/s across various regions/countries, status or maturity of telemedicine implementation, and various developments or changes that have been adopted during the COVID-19 are summarized. A comparative analysis of existing telemedicine frameworks using Gladhart and WHO/PAHO hat models, their stage, and recommendations from this consortium on improving telemedicine uptake during and beyond COVID-19 have been provided in Table 1.

**Table 1:** Status of telemedicine in different geographical regions and recommendations from the Consortium.

**Region**	**Pre-COVID-2019 status of telemedicine**		**Current status of telemedicine**		**Recommendations from Consortium**
	**Gladhart Model**	**PAHO Hat Model**	**Gladhart Model**	**PAHO Hat Model**	
Central Asia	Central Asian countries could follow the example set by Kazakhstanis in the adoption stage of telemedicine, in the process of implementing *Digital Kazakhstan* state program.	Kazakhstan has strategized programs to account for its large geographical stage, has formulated public policy, and is in the development phase of rolling out programs.	Kazakhstan was able to respond to the COVID-19 crisis through the adaptation of telemedicine and the establishment of telehealth headquarters.	Kazakhstan optimized telemedicine strategies with the backing of government support to provide primary and specialist care.	Countries with low population density could use Kazakhstan's focus on telemedicine as an example of how to provide far-reaching care. Nation-wide hospital networks and government support of telehealth startups could further telemedicine development.
China	China has varied degrees of implementation across telehealth realms. It demonstrates invention in novel technologies and population health management; however, some telemedicine regulations and implementation are still entry-level and requires further clarification.	China has begun to organize and develop telehealth networks, particularly in major cities such as Shanghai. However, due to large economic disparities between rural and urban areas, a further strategy is needed to avoid disparities in access to care.	COVID-19 has seen continued invention in China, with innovations such as the Wuhan AI hospital. Telehealth companies are also seeing increased adoption.	The Chinese government has taken a serious approach to COVID-19 management and continues to consider public policy and strategization in affected Chinese regions.	Clear regulatory telehealth frameworks would help facilitate the wide-scale application of telemedicine services. Collaboration between Asian regions is also needed to spread technological innovations.
Singapore	Singapore is encouraging invention through its regulatory sandbox, in terms of direct-to-patient telehealth platforms. Its National Framework has also allowed for adaptation from different hospitals and developers.	Singapore has fostered relationships between government, commercial developers, hospitals, and institutions to implement and optimize telemedicine strategies.	Relaxation of some regulations has allowed for the adaptation of telemedicine services by primary and specialist physicians. Continued invention and integration of telehealth apps and services have also seen improved patient care.	The continued development of regulatory support and integration of telehealth start-ups into the health system has demonstrated effective COVID-19 management solutions.	In a population-dense country such as Singapore, efforts to distance patients are paramount. With COVID-19 cases on the downturn, the focus should remain on encouraging innovation and consideration of international strategies such as mobile stroke units and artificial intelligence systems for Singapore.
Bangladesh	Bangladesh is in the entry stage of telemedicine progress, with no national telemedicine framework and lack of infrastructure.	Bangladesh hospitals do have telemedicine systems in place, with the utilization of multiple hubs and spoke structures taking place. Public policy planning is needed before further optimization can take place.	Limited analysis of telehealth implementation as a result of COVID-19 in Bangladesh is available.		The system will benefit from gaps analyses to ensure various aspects of telemedicine from infrastructure to privacy are adequately addressed
India	India is in the adoption stage of telemedicine, with governance bodies and guidelines in place, indicating the willingness of the government to implement telemedicine programs. However, ongoing issues such as limited infrastructure and lack of comprehensive regulation inhibit this process.	India needs to further establish a public policy to progress to the widespread development of telemedicine. Issues that require targeting include lack of clear policy, comprehensive guidelines, formats, or accountability frameworks.	Due to COVID-19, more patients have turned to telemedicine and hence pushed the adoption of telemedicine in more healthcare practices.	The Indian government issued new guidelines for telemedicine practice in March 2020. This step-in public policy aims to give further guidance to the practice of telemedicine in India.	To achieve widespread telemedicine in India, infrastructural and access issues must first be targeted. Furthermore, it must be ensured that comprehensive and binding legislation exists that provides clear physician-patient formats to cover issues of consent, privacy, and utilization.
Africa	The state of telehealth progress in Africa varies country-to-country. Countries with more advanced infrastructure and economic development, such as South Africa, are in the adoption and adaptation stages, whilst other LMIC is in the entry stage and require international support.	Broadly, strategic consideration of cultural, economic, and infrastructural needs is required to form a foundation for telehealth development in many African regions. Solutions to internet access and economic inequalities are needed for effective program development.	As COVID-19 cases begin to rise in Africa, support from other countries and the WHO is needed to assist with entry of the telemedicine support.	Public policy changes in countries such as South Africa reflect the need for expanded telemedicine capabilities during this time. Increased strategization is needed for countries with limited access to current telehealth.	Different regions of Africa require unique approaches, with some effective telehealth programs already in place. Infrastructure and government support of telemedicine partnerships are required to set the groundwork in many countries. International funding is required before local government funding will be sufficient.
Latin America	The level of telehealth in Latin America is mixed, with some examples of the invention, such as innovative telehealth partnerships. Increased adoption is needed across the population through the promotion of such platforms.	Many countries have a National Telehealth Strategy; however, national telehealth departments and clear regulations are required. Further public policy and development of telehealth infrastructure would be beneficial.	Increased adoption of telemedicine as a result of COVID-19 has been seen in some Latin American countries.	Public policy and promotion of telehealth platforms have been seen, but further work could be done on a governmental level to increase popularity.	Implementing change management strategies with the support of various stakeholders is required in this region. The penetration of commercial telemedicine solutions could also give more options to patients.
Caribbean	Many Caribbean countries are still in the entry phase of telemedicine.	Some telemedicine networks exist, and increasing levels of telehealth programs such as national EHR systems are eventuating. Identification of needs is needed for strategy, as are coordination teams for the organization.	Limited analysis of telehealth implementation as a result of COVID-19 in the Caribbean is available.		Basic telehealth programs are required in Caribbean countries. International models of technologies such as EHR systems may be a building block in this direction. Region-wide telehealth solutions through collaboration could also be advantageous.
Australia	Australia is in the invention stage of telemedicine progress. It needs to be ensured that these benefits extend to remote Australia, due to its vast land size and relatively isolated communities	Australia continues to seek to optimize and evaluate current telemedicine initiatives. Ongoing public policy relevant to disadvantaged communities and those with limited mobility/access is required.	Adaptation of current telehealth platforms is ongoing, and expansion to specialist care is recommended.	Governmental regulation changes have allowed for increased access to telehealth, through a comprehensive Medicare public health insurance Scheme.	Focus on specialist care delivery is required. Furthermore, collaboration with international think tanks and healthcare bodies is required for continual innovation in telemedicine delivery across the healthcare system.
New Zealand	New Zealand is in the invention stage of telemedicine progress, and further support of the technological sector is needed to bring it to the level of the likes of Australia and the USA.	New Zealand's Ministry of Health and dedicated National Telehealth Service allows for targeted telemedicine strategy.	The adoption of telemedicine has been promoted by clarifying guidelines and advice to healthcare practitioners.	Continued consideration of best practices and public policy surrounding telehealth is ongoing in New Zealand.	Support for innovation and international collaboration would be beneficial for New Zealand.
Canada	Canada could be considered as being in both the appropriation and invention stage of telemedicine progress, with levels of technological innovation combined with provincial differences in uptake.	Policy surrounding telemedicine and billing differs according to provinces. Government support of telehealth initiatives promises continued development.	Physician colleges and government guidelines seek to provide clarity and increase adoption during this time.	The COVID-19 pandemic has pushed the need for telemedicine development in Canada and highlighted the need to support urban regions as well as rural areas.	National cohesiveness across provinces and government promotion of the telehealth industry is useful.
USA	The USA is a frontrunner in telemedicine invention through hospital and technological innovations.	The USA continues to implement and optimize telehealth programs within and across states.	Government support and regulatory relaxations allow for increased penetrance of telemedicine across specialities.	COVID-19 has pushed the need for innovation within the telemedicine sector, and continued monetary support and funds allocation will help to continue this trajectory.	Disparities in health care insurance within the USA result in different levels of access for patients. It needs to be ensured that those patients who are immunocompromised, disadvantaged, immobile, and suffering from chronic care are receiving appropriate levels of support. American social platforms that are being utilized for teleconference means also need to ensure the privacy and security of their users.
UK	The UK is in the invention stage of telemedicine and continues to innovate with support from large telecommunication companies.	The UK continues to implement and optimize telemedicine services and has a governmental “Long Term Plan” in place.	Primary care, clinical trials, counseling, and chronic disease reviews are all rapidly being moved to telehealth delivery as different specialities increasingly adopt telemedicine.	Telehealth platforms continue to see increasing development and utilization as pressure from the COVID-19 pandemic pushes the need for remote services.	Ongoing governmental support of telemedicine innovation is necessary, and sharing of technological resources with other successful European healthcare systems such as France and Germany would be favorable.
Italy	Italy has mixed progress in telemedicine, with some areas being entry-level and others at adoption and adaptation. This is due to some deficiencies in the hospital infrastructure, supply-chain issues, and internet capabilities.	Italy requires public policy support of telemedicine, as it is not considered an essential item to patients within its National Health Service.	Italian hospitals became overwhelmed during the COVID-19 crisis. Hospitals and regions that were effectively available to adapt to telemedicine need to share their experiences.	Italian health care practitioners are sharing their experiences to identify needs and demonstrate the urgent need for telemedicine solutions.	This pandemic experience demonstrates the devastating impact of sudden surges in medical resources and personnel demand can have in the wake of unexpected disasters. Public policy support of telemedicine is needed in Italy, with successful hospitals sharing knowledge at a national level.
Spain	Spain is in both the entry and adoption stage of telemedicine, with progress varying in region to region.	Spain lacks a national telemedicine policy, which is required for the development of nation-wide effective telemedicine strategies.	Some hospitals have reported a rapid switch to telemedicine for outpatients and staff conferences by adapting current telemedicine technologies. Spain has also seen increased adoption of telehealth app usage.	There haven't been any notable policy changes to telemedicine in Spain as a result of the COVID-19 crisis. As with Italy, successful hospitals and regions need to share their successful strategies.	Precise governmental regulation and legislation that directly targets telemedicine are required in Spain. Furthermore, cooperation with other European countries would help to implement telemedicine models.

### Status of Telemedicine Services in East, Southeast, and South Asia

A 2016 multi-country study by Suzuki et al. assessed the possibility of introducing telemedicine services in several developing Asian countries (15). Indicators including healthcare environment, national information technology (IT) progress and economic status were used to assess the viability of internet-based medical services. It was found that countries such as Thailand, which have a shortage of physicians, but high levels of internet and mobile phone penetration, have a high possibility of telemedicine implementation. Despite high gross domestic product (GDP), countries like India and China face substantial variations in telemedicine uptake between urban and rural areas. Rural-urban gaps need to be reduced to leverage nation-wide telemedicine effectively.

Asian telehealth platforms are reporting drastically increased usages, such as Indonesian telehealth platforms Alodokter, Halodoc, and GrabHealth, driven by government support and recommendation (16). The Doctor Anywhere COVID-19 Medical Advisory Clinic has also been rolled out across several Asian countries such as Singapore, Thailand, and Vietnam, in which members of the public can undergo a video consultation through the app if they suspect they might have COVID-19 (17). Screening questions about symptoms, travel history, and close contacts will help determine if the person should admit themselves to the hospital. However, the level of subsequent support and payables differ between countries, with Singapore leading the way in ambulance-linkage and free consultation. Collaboration across Asian regions is needed, as well as bridging any access gaps within countries.

### Singapore

Singapore is a front-runner in Asia in terms of telemedicine adoption and healthcare system efficiency. In 2018, the Singapore Ministry of Health launched a regulatory sandbox to facilitate innovation and form relationships between the government and telehealth partners (18). In April 2019, the Health Science Authority released regulatory guidelines to clarify existing regulations around telemedicine and telehealth in Singapore, including categorization and risk stratification of services and regulatory controls surrounding medical devices (19). There are now 11 start-ups in the sandbox, with platforms such as MyDoc and DoctorAnywhere experiencing major upturns in use during COVID-19 (20). In April 2020, the Ministry of Health also announced that patients who qualify for Community Health Assist Scheme and MediSave, with a listed chronic disease, can now be covered to see their regular physicians through teleconference. However, this does not apply to new patients that have not yet had a physical consultation (21). In the context of COVID-19, Singapore Medical Association has issued specific guidelines and advice on the use of telemedicine during an infectious disease outbreak (22).

### India

Before 2020, telemedicine in India was governed by the IT Act, 2000, with gaps in clarity on privacy, security, and patient confidentiality, putting both patients and clinicians at risk (23). The Indian government issued new guidelines for telemedicine practice in March 2020 (24). Previously, a National Telemedicine Task Force was set up by the Ministry of Health (MOH) in 2005. A policy action toward digitizing healthcare was initiated by the Ministry of Health and Family Welfare of India (MoHFW) following the launch of the “Digital India” campaign by the Government of India in 2015. Later, in 2017, the National Health Policy (NHP) set forth goals to create an integrated health information system for all stakeholders in the health system, to improve efficiency, transparency, and citizen experience (25). The National Digital Health Blueprint (NDHB), released in January 2020, presents a detailed architectural framework of a “*Federated National Health Information System.”* It proposes to link systems within private- and public-health provider organizations across primary-, secondary-, and tertiary-care value chains (26). Another initiative by MoHFW is the start and delivery of online OPD services for all Indian citizens through the National Teleconsultation Service (eSanjeevaniOPD) (27). The eSanjeevani OPD- “Stay home OPD” will potentially improve accessibility and enable citizens to avail free health services through teleconsultations (27). More recently, on 15th August 2020, on the occasion of the 74th Indian Independence Day celebrations, the Prime Minister of India, Mr. Narendra Modi, officially launched the “National Digital Health Mission,” under the aegis of which every Indian citizen will be given a “Digital Health Card” containing all the information on their health issues, diagnosis, and relevant reports (28).

Telemedicine projects have been pursued by the Indian Space Research Organization (ISRO), Department of Information Technology, Ministry of External Affairs and MoHFW (29). However, these programs have been limited in their implementation and efficacy, barring few corporate hospitals that have developed and implemented their telemedicine networks (29). Despite the importance of telemedicine laid out in Digital India and NHP, telemedicine in India warrants a comprehensive strategy toward rapid implementation and scale-up. For example, the Telemedicine Pilot Project initiated by the ISRO has shown snail progress and uptake, connecting only 45 remote and rural hospitals and 15 super-speciality hospitals in 20 years (30). This has been attributed to limited infrastructure and lack of regulation (31).

Some of the national projects undertaken by the Ministry of Health and Family welfare are Integrated Disease Surveillance Project (IDSP), National Cancer Network (ONCONET), National Rural Telemedicine Network, National Medical College Network, and the Digital Medical Library Network (23). The framework developed by the MoHFW included five scenarios (32): (1) Patient to Registered Medical Practitioner; (2) Caregiver to Registered Medical Practitioner; (3) Health Worker to Registered Medical Practitioner; (4) Registered Medical Practitioner to Registered Medical Practitioner (31) and (5) Emergency Situations. The lack of clear policy and legislation has also deterred investments in telemedicine from the private sector. While a set of guidelines is available, it is neither comprehensive nor binding. There is no standardized format to qualify patient-physician interaction or to seek patient consent for privacy and confidentiality. Furthermore, there is no accountability framework to tackle medico-legal negligence matters or malpractice liabilities (31, 32). Sustained funding from the government to improve internet and telecommunications availability across the country, an extensive network of primary healthcare centers for service delivery, and a trained health workforce are critical to realizing NDHB proposal (33).

### Bangladesh

Currently, no national telemedicine framework in Bangladesh exists; and current systems suffer from lack of technological infrastructure, healthcare inequities, and poor treatment quality (34). The current system is modeled on multiple hubs and spokes where consultations and training are provided between providers over video conferencing using Skype. The sub-district hospitals can consult district hospitals, which in turn can connect to central specialized hospitals. The system will benefit from gaps analyses to ensure that various aspects of telemedicine from infrastructure to privacy are adequately addressed. No comprehensive telemedicine efforts to boost capacity have been pursued amidst COVID-19.

### China

China has a sizeable urban-rural health gap, which major telehealth networks such as the International MedioNet of China network, Golden Health Network, and the People's Liberation Army Telemedicine Network have not been able to rectify (34). In September 2018, the National Health Commission and the National Administration of Traditional Chinese Medicine released new e-healthcare rules to expand telehealth capabilities and develop the telehealth industry. This included guidance around a collaboration between commercial companies and hospitals, telehealth diagnosis, patient consent, and third-party collaborations (35). This broadened telemedicine definitions beyond physician-to-physician consultations to include physician-to-patient interactions, but still lacks some clarity in implementation and regulation (36). Like other Asian countries, online healthcare platforms are projected to see drastic increases in market share in the coming years, particularly following COVID-19, with companies supplying direct-to-patient telehealth subscriptions such as Good Doctor, Alibaba, and Tencent experiencing growth (36). China is also breaking new ground in terms of contactless innovations, such as a Smart Field Hospital trial in Wuhan during COVID-19, in which patient care is delivered through robots and digital devices (37).

### Central Asian Region at a Glance-Focus on Kazakhstan

Kazakhstan implemented a national telemedicine network (NTMN) in 2004 (38). Its purpose was to eliminate the gap in the availability of specialized medical care for the urban and rural populations of the remote areas. This telehealth network combines 199 healthcare objects of districts, regions, and republican organizations (38). Patients in regional and urban district hospitals receive teleconsultations from the doctors of regional hospitals and republican clinics of Almaty and Nur-Sultan cities in Kazakhstan. More than 500,000 telehealth consultations have been provided via video conferencing over the last 15 years. The first large-scale project in Kazakhstan, “*Development of telemedicine in rural health care*,” was launched in 2005 within the framework of the state program (39). During a typical session, doctors send the patient's data such as ultrasound, electrocardiography, X-rays, laboratory tests to the specialist consultant of the telemedicine center. The medical consultant gives his view for the diagnosis and recommendations for an additional examination and treatment. In 2018 a 5-year state program “*Digital Kazakhstan*” was run. Five critical areas, including medicine, are planned for digital transformation (40). As a part of the program, for the last 2 years, unified electronic medical records and doctor appointment mobile application systems have been implemented all over the country. In Kazakhstan, telemedicine is especially essential due to the nation's vast geographical spread and low population density. It is becoming increasingly crucial and being expanded to rural areas, 100–500 kilometers away from the city. In the COVID-19 era, telemedicine centers organize video consultations of patients with COVID-19 by sub-specialists, if necessary. The first COVID-19 case in Kazakhstan was confirmed on March 13, 2020, and more than 1,200 new cases have been confirmed to this date (41). At present, patients diagnosed with COVID-19 are being treated at regional hospitals. As soon as the first messages about COVID-19 were received, administration of the East Kazakhstan region established telehealth center headquarters at the premises of Semey Medical University (42). This experience was shared with other regions. The purpose of the headquarters is to limit exposure to physicians allowing doctors to consult with patients *via* videoconferencing. There is a mobile tablet at the patients' wards, which enables them to carry out visual monitoring and assessment of the patient's condition. The doctor who treats a patient in the department may request the consultation of their colleagues *via* videoconferencing, who are members of the headquarters, including: emergency physicians, pulmonologists, or infectious disease specialists. Telehealth enables access to consultations of any specialized health professional and, consequently, ensures the ensures the quality of care provided to the patients while avoiding exposure of the medical staff to potential CVOID-19 contagion. The positive experience of telehealth-laden headquarters has led to the Ministry of Health of the Republic of Kazakhstan and WHO proposing this model to be used in other regions of the country (42). Kazakhstan sets an example to other countries with a large geographical area but low-density populations in optimal use of telehealth platforms during COVID-19.

### Spotlight on Africa

Sub-Saharan Africa is a region of swift telehealth development, as program implementation increases, and smartphone penetrance is expected to reach 66% by 2025 (43). This region suffers from the world's highest burden of disease and shortage of health workers, as well as barriers to telehealth implementation such as connectivity issues, limitations in device ownership, language barriers, high costs, and electricity access. This highlights the necessity for building supporting infrastructure, government support and involvement, upskilling of the workforce, and legislative compliance. Suzuki et al. reported that in countries such as South Africa, Egypt, Morocco, and Algeria, high internet and mobile penetration rates open up the possibility for telemedicine to compensate for large physician shortages (15). Economic development, mobile phone penetrance, and internet speed all need to be considered when considering future telemedicine possibilities. A systematic review of initiatives in Kenya showed that there were several eHealth projects, mostly focused on primary care and HIV/AIDS, but vast inequalities existed between more central and remote parts of the country in terms of delivery (44). In other African countries, such as Burkina Faso and Nigeria, lack of political support may be a cause of slow implementation (45). In 2014, the Health Professions Council of South Africa (HPCSA) released guidelines to deliver healthcare to underserved communities.

Telemedicine is still in its infancy in Africa due to ongoing conflicts and war, inadequate information communication technology (ICT), and lack of funding and political support for technology diffusion and policy (46). In light of the COVID-19 pandemic, disagreement between the HPCSA and the South African Medical Association ensued over matters such as the HPCSA's adversity to widespread telemedicine replacement over face-to-face consultations and requirement for previous face-to-face doctor-patient consultation (47). This resulted in amended guidelines in early April 2020. Africa in general, Sub-Saharan Africa in particular, suffer a high burden of disease and physician shortages and hence require international support to assist instigation of digital health efforts (48, 49). Digital health innovations such as mobile technologies offer a cost-effective strategy to improve health service delivery in Africa (50).

### Focus on Latin America

Latin America has one of the fastest-growing elderly populations worldwide and has a big divide in access to healthcare providers between rural and urban regions (51). A 2019 study analyzed telemedicine expansion capabilities across 9 Latin American nations (52). It reported that since 2014, certain countries, including Peru, Colombia, Guatemala, Panama, Uruguay, Mexico, Costa Rica, Chile, and Argentina have developed a national telehealth strategy; other countries such as Panama, Mexico, and Guatemala do not have a national policy. It also reported an imbalance between public and private hospitals, with telehealth adoption in public hospitals being 30% higher. Notably, Chile has the highest telemedicine uptake in the region and has successfully transitioned from international funding to government funding (52). Innovative inter-country programs for myocardial infarction treatment have been set up, which facilitates linkages between patients in Columbia, Mexico, and Brazil through a centralized network (53). The Enlace Hispano-Americano de Salud (EHAS) Foundation also seeks to provide healthcare to rural Latin American communities through technology (54). It researches into communication networks, and how-to bring infrastructure to underdeveloped regions, through means such as low-cost Wi-Fi solutions (54). In 2006, a Telemedicine University Network (“Rute”) was set up in Brazil that connects 124 universities and teaching hospitals-making it, to our best knowledge, the biggest telemedicine/telehealth program in the world (55). Rute has allowed healthcare providers in Brazil to connect with their peers around the world, especially Portuguese speaking countries.

Targeted government support of telemedicine is needed in this region, as telemedicine in countries such as Mexico falls under the standard practice of medicine regulations, instead of being governed by a focused telehealth department (56). In 2018, the United Telemedicine Network was established in the Dominican Republic as a private network (57). As a result of COVID-19, telehealth is becoming increasingly popular in the region, with countries such as Brazil relaxing regulations for the duration of the pandemic. However, countries such as Argentina are still slow on the uptake of telemedicine (58). Overall, telemedicine adoption is becoming more widespread in Latin America, but certain regions need work before its full potential could be realized. This could be possible by implementing change management strategies with the support of various stakeholders.

### Focus on the Caribbean

Telehealth in the Caribbean is in a relatively early stage, with a lack of cohesive telehealth strategies and policies. The National Health Authority in the Bahamas announced a new Electronic Health Record (EHR) system at the end of 2019 (59), which reduces the need for paper records and allows the effortless transfer of patient care, whilst the Dominican Republic is yet to have a national EHR system (60). Some burgeoning initiatives include a 2018 Telemedicine Pilot Project initiated by the Jamaican Minister of Health in the West Indies and the SickKids-Caribbean Initiative (SCI) that uses telemedicine to provide medical services for children with cancer and blood disorders across the Caribbean region (61, 62). Ongoing work is needed in this region to bring telehealth solutions to the broader population especially those from remote locations.

### Focus on Australasia

#### Australia

Australia is in the process of making telehealth implementation more accessible for providers since the COVID-19 outbreak (63). New Medicare Benefits Scheme (MBS) items will allow access to telehealth services to all Australians with a Medicare card, and bulk-billing for all concession card holders (63). The MBS is a list of the medical services for which the Australian government pays Medicare rebates. Expansion of telehealth beyond primary care to specialist care with the help of relevant medical colleges and societies is currently being planned (64).

#### New Zealand

The Ministry of Health in New Zealand has released an advisory on available teleconsultation technologies for health care practitioners and best practices around patient privacy (65). The Medical Council of New Zealand has also released guidelines, particularly around prescription practice and the need for face-to-face consultation, whether that be in-person or via teleconference, before writing any scripts (66).

### Focus on Europe and North America

#### Europe

The threat of COVID-19 in Europe has necessitated the need for telehealth platforms readily available to patients. Telehealth startups such as *French Doctolib* and *Qare*, Swedish LIVI, UK's Push Doctor, and Germany's Compugroup Medical SE have all seen considerable increases in European uptake numbers (67). In Europe, telemedicine is considered both a health service (Directive 2011/24/EU) and an information service (Directive 95/46/EU, Directive 2000/31/EC, and Directive 2002/58/EC). Due to lack of pan-European uniform medical liability and medical legislative regulations, a Europe-wide framework is far from being realized (68).

#### UK

In the United Kingdom (UK), telecommunication companies such as BT, Virgin Media, and Sky in the United Kingdom have agreed to support the National Health Services (NHS) in rolling out telehealth for healthcare practitioners (69). Primary care, clinical trials, counseling, and chronic disease reviews are all rapidly being moved to telehealth delivery (70). Prior to COVID-19, the government had already announced a centrally funded “Long Term Plan” to reduce the number of outpatients presentations (71). This had to be put into place much faster than anticipated as the need for telehealth to replace outpatient visits has drastically increased.

#### Italy

It has been reported that many Italian hospitals lack the infrastructure for effective telehealth platforms, due to supply-chain breakdown and insufficient internet capabilities (72). Italy also does not include telemedicine as an essential item to patients within its National Health Service (11). This is despite the implementation of telemedicine guidelines by the Italian Health Council in 2012, aimed at increasing telehealth penetration (73). Being one of the first countries to become overwhelmed by COVID-19, Italy was unprepared for the surging requirement of medical resources and personnel, and Italian physicians are urging other countries to avoid hospital-acquired transmission, wherever possible (74). No advisory on telemedicine was provided by the Italian health authorities until March 24 (75, 76), when an open call for telemedicine and monitoring system technologies were made by the Italian Health Ministry and Ministry for Technology Innovation and Digitalization jointly with the WHO (77).

#### France

The telemedicine regulations in France developed before COVID-19 allowed tertiary and primary care physicians to switch scheduled in-person consultations to teleconsultations, as and when appropriate (11). All video consultations and tele-expertise were allowed reimbursement, for those with COVID-19 or with COVID-19 symptoms, by the French National Health Insurance (NHI) by a decree signed by the Ministry of Health on March 9, 2020, without the requirement of the patient being known to providers beforehand (78). This change was aimed to reduce exposure to providers and patients, and also to identify patients who are likely to be COVID-19 positive. On March 19, 2020, additional funding for follow-up *via* video or phone consultations by nurses were provided. This was expanded to include teleconsultations by speech therapists on March 25, 2020.

#### Spain

Spain, like Italy, has been overwhelmed by the current pandemic, and tertiary hospitals have had to adopt new telemedicine strategies rapidly. In 2012, Spain's national health authority launched a health IT strategy; however, it does not have a national policy that directly implicates telemedicine (79). To some extent, telemedicine adoption varies from region to region in Spain. Hospitals such as *Denia Marina Salud* Hospital have reported a rapid switch to telemedicine for outpatients and staff conferences to good effect (80). Interestingly, Spain's leading telehealth app, MediQuo, has now made consultations regarding COVID-19 free of charge (81).

#### United States

In the COVID-19 era, Medicare in the United States will now cover eligible telehealth consultations, and individual states are being encouraged to roll out Medicaid cover for such services (82, 83). Several commercial insurers have also followed suit in paying healthcare providers for telehealth services or are directly providing telehealth access to their members as part of benefits. The relaxation of prior regulations also includes an expansion of covered telehealth services and allowing new patients to be treated through telemedicine rather than only those with a prior relationship (84). Penalties for HIPAA violations for telemedicine are currently waived in the face of the COVID-19 pandemic when HCP are acting in good faith. Additionally, to facilitate telemedicine, practitioners licensed out of state and in good standing are allowed to practice across state lines. Furthermore, the Federal Communications Commission recently launched a $200 million Coronavirus Telehealth program (85). One of the challenges in the adoption of telemedicine has been the variations in uptake and differential policy frameworks across the different states. These variations merit a federal telemedicine policy initiative to harmonize and accelerate an overarching framework which would potentially improve its penetration across the United States.

#### Canada

In Canada, the availability of telehealth varies from province to province (86). A particular issue has been that telemedicine technology has primarily been allocated to rural services in the past, where now urban centers have a sudden need for them (87). However, the government has sought to rectify this by promising to increase the telehealth capabilities of healthcare facilities. The Royal College of Physicians and Surgeons of Canada has also put together a guideline for physicians from each province, outlining in which situations to use telemedicine and any changes to billing codes (88).

### Telemedicine/Telehealth Application in Mental Health

Populations in LMICs, particularly those impacted by war, experience disproportionate levels of mental health burden compounded by lower levels of mental health treatment (89). Telehealth programs such as public health campaigns to reduce stigma and telehealth programs through mobile phones have shown effectiveness in Afghanistan and may be implemented in other LMICs (90). E-health, or digital health, in which mental health information can be sourced online, has implications for countries worldwide, as mobile phones are becoming increasingly prevalent. Individuals often go online to find information about mental illness, and hence digital platforms such as digital informative programs, not-for-profit organizations, and free helplines should receive ongoing international support (91).

### Telehealth in Public Health Response and Community Engagement

In a public health crisis, telehealth could be a great tool to foster community engagement. Through targeted education programs aimed at training community leaders from underserved populations, telehealth could help to expand access to health care providers in remote areas. It could be used to provide medical services, including allied health services, and ongoing professional development of students and staff at remote healthcare facilities. Clinical supervision and mentoring could also use telehealth infrastructure (92). The coronavirus pandemic has shown that continuous learning, or rather e-learning is not only possible, but very effective, and can include a much wider group of people (93).

### Telemedicine for Unstable Regions

A potential role of telemedicine can be realized in areas affected by war, ongoing violence and conflict, terrorism, and natural disasters (94–96). People inhabiting these regions suffer from an acute lack of basic medical provisions and access to health services. Telemedicine can complement and enhance the capacity of medical humanitarian agencies, organizations, or missions such as *Doctors Without Borders*. An international teleintensive care unit program was launched in 2012 to provide medical services in war-torn Syria (95). This model, leveraging telemedicine and artificial intelligence could also be deployed in other unstable regions to mitigate infectious disease vulnerability (IDV) in resource-limited settings such as the African countries (especially those in a civil war-like Burkina Faso) that scored lower than the average global health security (40.2) index. This could be important in building global health security while maintaining critical access and sustainability of health systems.

### Language Barriers in Telemedicine in LMICs

Language barriers are associated with risks to patient safety and quality of care. They can pose a significant barrier in telemedicine uptake, specifically among culturally and linguistically diverse populations across low-resourced settings and developed regions alike, affecting providers and patients alike (9, 97–106). Communication barriers have been shown to impact patients adversely. With an increasingly diverse demographic makeup, patients from culturally and linguistically diverse backgrounds (CALD) or ethnic backgrounds are at increased risk of widening disparities in healthcare access and quality (107). Moreover, this population also consists of uninsured migrants or refugees who have limited knowledge of healthcare services and diseases that could be risk-managed (101, 107). Language barriers could potentially exacerbate the structural disparities, and therefore, telemedicine approaches should consider resources or technologies that could provide appropriate language support to the vulnerable communities (32).

## Discussion

**Figure 1:**
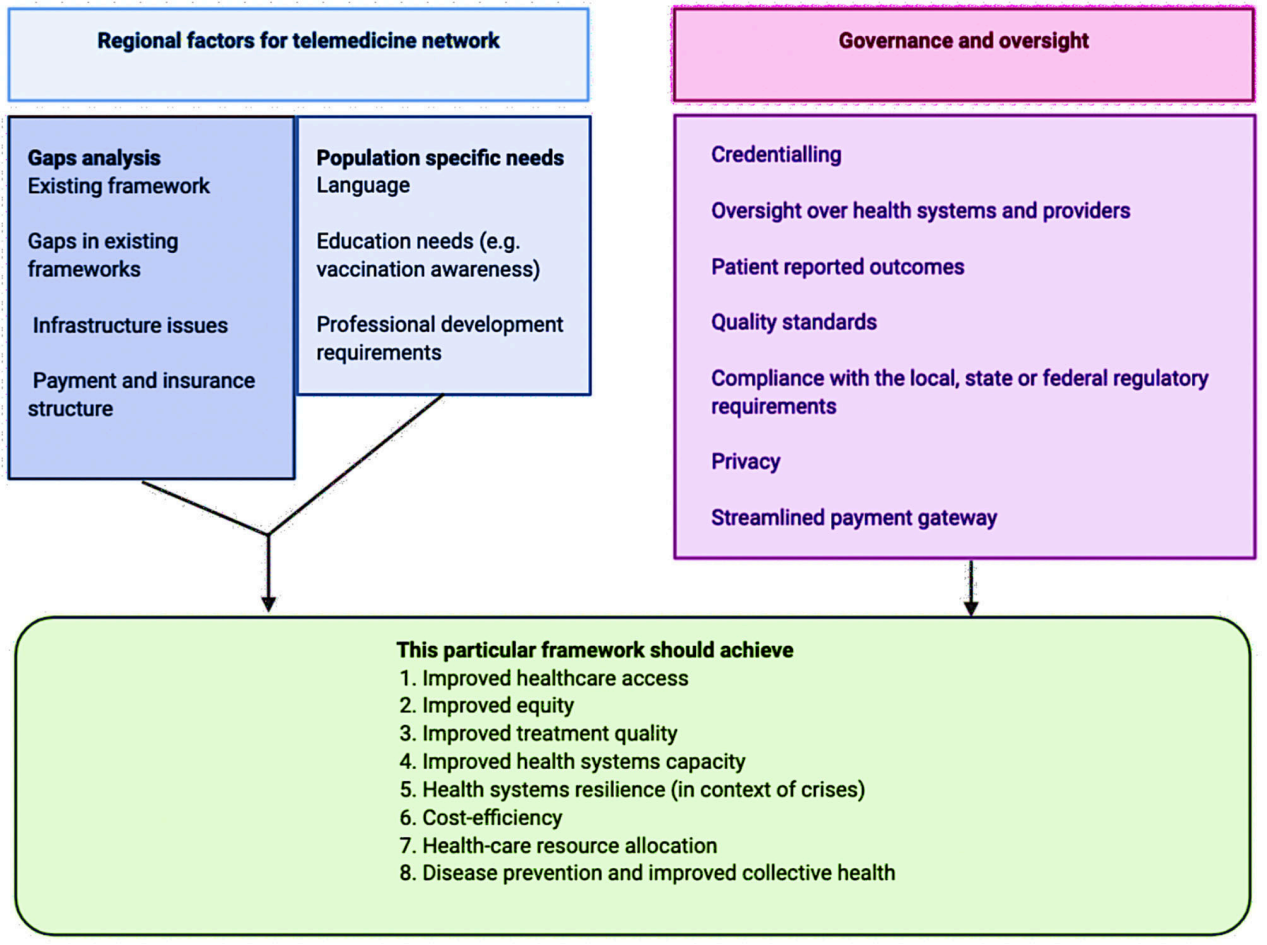
Various regional and structural factors in telemedicine framework development and implementation.

Public health crisis, such as COVID-19, severely challenges the health systems (5, 7, 11). In this paper, we have critically examined the current state of telemedicine across geographics and the various steps taken amidst COVID-19 for its uptake and implementation. Some of the identified challenges/barriers and potential benefits to stakeholders are summarized in Figure 1. There is a compelling need for resources within individual states, countries, and regions to harmonize policies to establish an international framework on telemedicine (108). The consensus on uniform standards for telemedicine practices is missing. International organizations, including WHO, International Monetary Fund, World Economic Forum, and current consortium, could take the lead in facilitating such initiatives. The recommendations and considerations made by this consortium are to facilitate a policy-based international debate on the integration of telemedicine within the public health preparedness and response strategy during outbreaks (Table 1). We postulate that by identifying the gaps in policy, framework, and implementation, while considering various structural barriers and interfering factors, telemedicine would act as a “safety net” while mitigating the devastating impact of outbreaks such as COVID-19.

Regional frameworks that allow for collaboration and sharing of resources need to be strengthened. In pandemic settings, when existing health systems are under enormous stress, open platforms for connectivity to use telemedicine and corresponding legislations to accommodate such provisions, so that telemedicine facilities could be readily available, in a secure manner while maintaining confidentiality and privacy in the doctor-patient relationship (33). Data sharing could also accelerate research programs by harnessing the data thus gathered. Commitment to equal access to quality care to all and digital health is a critical enabler for the overall transformation of the digital health ecosystem. Some barriers, such as technology and infrastructure specific to telemedicine in remote settings, including language barriers, need priority attention. Telehealth access is dependent on broadband access, smartphone usage and the presence of basic digital skills. A systematic review on the evaluation of barriers to telemedicine adoption worldwide found technology-specific barriers and lack of digital literacy as major impediments in telemedicine implementation (9). Not acknowledging and addressing these factors can further increase the digital disparity and lack of care access to those who may need it the most. This could be addressed through training, alternating in-person and telemedicine consultations, policy, and legislative changes, and change management strategies that consider local and cultural factors (9, 98). Digital literacy is a major barrier to telemedicine during COVID-19 (109).

Mainstreaming telemedicine in health systems could reduce healthcare inequities (110). Digital health is fast emerging as one of the most defining trends of this decade and will have a profound influence on geopolitical and socioeconomic realities in the future (111). Policies advocating the use of digital tools should be implemented that lays significant focus on the use of telemedicine services, especially in the Health and Well-ness Centers at the grassroots level wherein a mid-level provider/health worker can connect the patients to the doctors through technology platforms in providing timely and best possible care. This would improve efficiency, and clinical outcomes while also reducing costs of the healthcare system.

Therefore, for several countries, the fast implementation of telemedicine tools is still laborious and even impossible currently. Therefore, joint initiatives, like this consortium, might be instrumental in helping the governments to propose concrete solutions and policy directives to have these systems available. Despite advances in telemedicine and urgency in its deployment during COVID-19, people from vulnerable populations and/or from geographically disparate regions who have limited access to technologies may be at a disadvantage. Not all clinical examinations can be performed through telemedicine, and therefore, various clinical subspecialties need to develop guidelines for disease-specific telemedicine consultation programs complementing in-person appointments (7). Language and socioeconomic barriers need to be addressed along with maintaining patient continuity during COVID-19, including the need for in-person consultations when it is necessary.

An action plan centered around a global effort to integrate telemedicine into public health response to disease outbreaks such as COVID-19 is required (11). This would require embedding telemedicine as an integral part of national and international guidelines on health system preparedness (112). This along with international standards of interoperability can help widen telemedicine base (9). Barriers pertinent to digital divide needs to be addressed through targeted communication and engagement in respective languages on access and use of telemedicine. This has to be complemented with rigorous evaluation of telemedicine penetration and impact during public health emergencies or disease outbreaks. Beyond acute care, telerehabilitation is an important consideration, allowing long-term monitoring of patients who may require ongoing treatment and/or rehabilitation, especially those living in remote areas, where provision for traditional rehabilitation may be limited or relatively less accessible (113).

## Conclusion

In conclusion, despite the variabilities in telemedicine uptake across the world; increasing evidence suggests a positive role for telehealth or telemedicine in improving health systems performance and outcomes in developed countries (114). In the COVID-19 era, telemedicine has taken a springboard trajectory, as governments and partnerships, thus made, have forged a way to support and accelerate its roll-out especially in developed countries. Telemedicine during COVID-19 has demonstrated organizations' ability to deliver quality care remotely (at home) while also reducing costs (115). This is likely to be sustained beyond the COVID-19 era with strengthening and widening of telemedicine services. However, in resource-constrained settings, a significant number of structural issues remain which have been exacerbated due to COVID-19. Moving further, one particular area warranting attention is improving telemedicine use among sub-specialities, complementing primary care. There is an emerging role of building partnerships between various stakeholders and promoting open innovations to allow benefits of telemedicine/telehealth to reach the disadvantaged populations/geographics (116). Regional efforts such as pan-European initiatives may be explored around appropriate regulation and governance frameworks once the COVID-19 period ends. International efforts toward developing public health preparedness framework, with telemedicine at the core of public health response during outbreaks such as COVID-19, are warranted.

## Author's Note

The COVID-19 pandemic is causing an unprecedented public health crisis impacting healthcare systems, healthcare workers, and communities. The COVID-19 Pandemic Health System REsilience PROGRAM (REPROGRAM) consortium is formed to champion the safety of healthcare workers, policy development, and advocacy for global pandemic preparedness and action.

## Author Contributions

SBh devised the project, the main conceptual ideas, including the proposal for a new telemedicine workflow, the proof outline, and coordinated the writing and editing of the manuscript. SBh and SBr wrote the first draft of the manuscript. SBh encouraged SBr to investigate and supervised the findings of this work. All authors discussed the results and recommendations and contributed to the final manuscript.

## Conflict of Interest

SP is the Vice President of Immersive Medicine at Luxsonic technologies, a medical technology company specializing in virtual/augmented reality for medical education, collaboration, and training. AAt is the Scientific Founder and Board Member of Rx.Health, and founding chair of NODE.Health (Network of Digital Evidence in Health). The opinions expressed in this article are those of the authors and do not necessarily represent the decisions, official policy, or opinions of the affiliated institutions. The remaining authors declare that the research was conducted in the absence of any commercial or financial relationships that could be construed as a potential conflict of interest.
